# Management of drug-resistant TB in patients with HIV co-infection

**DOI:** 10.7448/IAS.17.4.19508

**Published:** 2014-11-02

**Authors:** Graeme Meintjes

**Affiliations:** Institute of Infectious Diseases and Molecular Med, University of Cape Town, Cape Town, South Africa

## Abstract

The World Health Organization estimates that 450,000 cases of drug-resistant (DR) tuberculosis (TB) occurred worldwide in 2012. In South Africa, over 15,000 cases were diagnosed. Over half of patients in South Africa with TB are HIV co-infected. The management of drug-resistant TB is complex, prolonged, costly, associated with multiple toxicities and thus difficult for patients to complete. Disengagement from follow-up is common. Co-infection with HIV presents a number of additional challenges in DR TB management including shared drug toxicities between TB and HIV drugs, potential for increased drug toxicity due to underlying HIV-related organ disease such as nephropathy, pharmacokinetic drug–drug interactions and immune reconstitution inflammatory syndrome including manifestations at extrapulmonary sites. Mortality with multi-drug-resistant (MDR) TB is higher in HIV-positive patients. Mortality is similar for HIV-positive and uninfected patients with extremely drug-resistant (XDR) TB, given the current lack of effective therapy, with over 70% case fatality by five years. ART improves survival in patients with DR TB, and timing of ART initiation in relation to TB treatment should be similar to patients with drug-susceptible TB. New (e.g. bedaquiline and delaminid) and repurposed (e.g. linezolid and clofazamine) drugs promise to improve the prognosis of patients with DR TB. Several clinical trials of new regimens are ongoing and planned, and early data from the Bedaquiline Clinical Access Programme in South Africa suggests much improved short-term outcomes when bedaquiline +/− linezolid and/or clofazamine are included in the regimen of patients with XDR and pre-XDR TB including patients with HIV co-infection. There are important considerations with respect to QT prolongation and ART drug interactions related to bedaquiline that need to be factored in treatment decisions and monitoring plans.

